# Editorial: Activated Synapses

**DOI:** 10.3389/fnsyn.2022.875904

**Published:** 2022-03-18

**Authors:** F. Javier Rubio, Emmanuel Valjent, Bruce T. Hope

**Affiliations:** ^1^Behavioral Neuroscience Research Branch, Neuronal Ensembles in Drug Addiction, NIDA Intramural Research Program, NIH, Bethesda, MD, United States; ^2^IGF, Univ. Montpellier, CNRS, Inserm, Montpellier, France

**Keywords:** neuronal ensembles, cellular engrams, synaptic engrams, long-term memory, drug of addiction

This Research Topic focus on synaptic alterations and synaptic engrams as they relate to ensembles mediating different learned behaviors, including those involving drugs of abuse. It is now widely known that small subsets of sparsely distributed neurons, called neuronal ensembles, play causal roles in mediating specific learned associations underlying learned behaviors. Different memories can be mediated within the same brain area by different ensembles. However, while the cell bodies that make up neuronal ensembles are critical integrators of information-specific input, the primary mechanism for mediating and storing long-lasting memories are thought to be encoded by learning-specific alterations within a specific pattern of synapses on these ensemble neurons. Accordingly, there has been a recent shift from focusing on the alterations of ensemble cell bodies (cellular engram) to alterations of ensemble synapses (synaptic engram). Hence, for the purpose of this Research Topic on Activated Synapses, we have gathered three reviews and one original research article. They are focused on glutamatergic and/or dopaminergic synaptic adaptations contributing to the formation of synaptic engrams and what we called activated synapses.

Behavioral effect of drugs of abuse such as psychostimulants are ultimately mediated by the integration of glutamate and dopamine inputs at the dendritic spines of striatal medium-sized spiny neurons (MSNs). Understanding how these two inputs integrate to activate synapses and produce long-lasting synaptic alterations that combine and trigger the final activation of a neuron are important biological questions that may be addressed using molecular and cellular techniques. Allichon et al. used a strategy for studying synaptic adaptations induced by dopamine and glutamate signaling and its effects on dendritic spine and synapse by manipulating cell-type-specific striatal MSNs expressing dopamine D1 or D2 receptors. In that direction, original research from Clerke et al. showed output-specific adaptation of lateral habenula-midbrain (LHb-to VTA) glutamatergic synapses during cocaine withdrawal. This study indicates that the LHb anatomically and functionally controls distinct subpopulations of VTA neurons. The rewarding and aversive effect of cocaine can be explained by a differential modulation of synaptic plasticity within LHb-to-VTA circuits during abstinence of drug intake, as well as by specific VTA cell subpopulations that project their axons to medial prefrontal cortex (mPFC) and nucleus accumbens (NAc). Therefore, not only cell-type but synapse-type are contributing to the effect of cocaine in those brain circuits. Synapses that are activated during learning are thought to undergo long-lasting alterations to form a long-lasting engram encoding the memory. In cortical neurons that are activated during learning and shown to be required for long-term memory expression, Rao-Ruiz et al. describe possible mechanisms for how structural synaptic changes such as clustered spine formation, spine size and density can act as synaptic engrams that underlie drug-associated memories. Most of the previous data about how these synapses are activated were obtained from neuronal cell culture or in slice preparations. However, the meaning of those changes and its translation to the brain system and the process of learning and memory is a challenge for the synaptic neuroscience field. To this end, Rao-Ruiz et al. discuss long-term alterations of cell and synaptic engrams that allow memory retention for up to 1 month following activation during learning.

New advances in molecular, cellular, and imaging techniques and experimental designs for studying activated synapses *in vivo* have recently become available. These include the isolation and identification of markers of synaptic activity (e.g., by using RNAseq and proteomic broad spectrum analysis), and the use of genetic tools for visualization and modification of those activated synapses. A candidate marker of activated synapses emerges from the discussion in the review by de Pins et al. about the potential role of a calcium-activated protein tyrosine kinase Pyk2 as a broad sensor of Ca^2+^-regulation in the dendritic spine.

Further studies in this direction will help us understand the process of memory and eventually develop targeted genetic and pharmacological approaches to differentially strengthen or weaken specific sets of synapses or synaptic engrams with the aim of consolidating or erasing memories underlying pathological or addictive conditions.

## Author Contributions

FJR contributed to conception of original proposal of the Research Topic and wrote the first draft of the Editorial. FJR and EV invited possible contributors and handled manuscripts to be reviewed. BH contributed to writing original proposal and Editorial. All authors contributed to original proposal, Editorial revision, read, and approved the submitted version.

## Funding

This work was supported by NIDA Intramural Research Program (to FJR and BH).

## Conflict of Interest

The authors declare that the research was conducted in the absence of any commercial or financial relationships that could be construed as a potential conflict of interest.

## Publisher's Note

All claims expressed in this article are solely those of the authors and do not necessarily represent those of their affiliated organizations, or those of the publisher, the editors and the reviewers. Any product that may be evaluated in this article, or claim that may be made by its manufacturer, is not guaranteed or endorsed by the publisher.

