# Corrigendum: Editorial: Activated Synapses

**DOI:** 10.3389/fnsyn.2022.932503

**Published:** 2022-06-23

**Authors:** F. Javier Rubio, Emmanuel Valjent, Bruce T. Hope

**Affiliations:** ^1^Behavioral Neuroscience Research Branch, Neuronal Ensembles in Drug Addiction, NIDA Intramural Research Program, NIH, Bethesda, MD, United States; ^2^IGF, Univ. Montpellier, CNRS, Inserm, Montpellier, France

**Keywords:** neuronal ensembles, cellular engrams, synaptic engrams, long-term memory, drug of addiction

In the original article, the funder NIDA Intramural Research Program to Bruce T. Hope and F. Javier Rubio was mistakenly not included.

The authors apologize for this error and state that this does not change the scientific conclusions of the article in any way. The original article has been updated.

## Publisher's Note

All claims expressed in this article are solely those of the authors and do not necessarily represent those of their affiliated organizations, or those of the publisher, the editors and the reviewers. Any product that may be evaluated in this article, or claim that may be made by its manufacturer, is not guaranteed or endorsed by the publisher.

